# Growth Inhibition and Membrane Permeabilization of *Candida lusitaniae* Using Varied Pulse Shape Electroporation

**DOI:** 10.1155/2015/457896

**Published:** 2015-12-01

**Authors:** V. Novickij, A. Grainys, E. Lastauskienė, R. Kananavičiūtė, D. Pamedytytė, A. Zinkevičienė, L. Kalėdienė, J. Novickij, A. Paškevičius, J. Švedienė

**Affiliations:** ^1^High Magnetic Field Institute, VGTU, Naugarduko 41, LT-03227 Vilnius, Lithuania; ^2^Department of Biotechnology and Microbiology, VU, M. Čiurlionio 21, LT-03101 Vilnius, Lithuania; ^3^State Research Institute Centre for Innovative Medicine, Žygimantų g. 9, LT-01102 Vilnius, Lithuania; ^4^Laboratory of Biodeterioration Research, Nature Research Centre, Akademijos Street 2, LT-08412 Vilnius, Lithuania

## Abstract

*Candida lusitaniae* is an opportunistic yeast pathogen, which can readily develop resistance to antifungal compounds and result in a complex long-term treatment. The efficient treatment is difficult since structure and metabolic properties of the fungal cells are similar to those of eukaryotic host. One of the potential methods to improve the inhibition rate or the cell permeability to inhibitors is the application of electroporation. In this work we investigated the dynamics of the growth inhibition and membrane permeabilization of* C. lusitaniae* by utilizing the various pulse shape and duration electric field pulses. Our results indicated that single electroporation procedure using 8 kV/cm electric field may result in up to 51 ± 5% inhibition rate. Also it has been experimentally shown that the electroporation pulse shape may influence the inhibitory effect; however, the amplitude of the electric field and the pulse energy remain the most important parameters for definition of the treatment outcome. The dynamics of the cell membrane permeabilization in the 2–8 kV/cm electric field were overviewed.

## 1. Introduction

The* Candida* species are commensal yeasts in healthy humans and are considered the causative agent of opportunistic fungal infections [1, 2]. The mortality associated to* Candida* infections is near 40%, making candidiasis a major health problem in immunocompromised cases [1, 3, 4]. In clinical specimens* C. albicans* is the most pathogenic and commonly encountered yeast; however, recent reports indicate a trend towards an increasing prevalence of infections caused by nonalbicans* Candida* species [5, 6]. Nonalbicans* Candida* species are emerging as both colonizers and pathogens causing nosocomial fungal bloodstream infections [7, 8]. The biofilm-associated infections, when the cells form highly organized heterogeneous clusters embedded in polysaccharide matrix, are particularly dangerous and difficult to treat due to the increased resistance of the biofilm to the antifungal drugs [6, 9]. However, even though the drug resistance of the biofilms is complex and depends on many factors, the research of new antifungal methods causing inhibitory effects is constantly performed [2, 10]. An ideal antifungal agent should have a wide spectrum antifungal activity and must not cause toxicity to the host [11]. Currently, a limited number of antifungals such as amphotericin B, fluconazole, itraconazole, and voriconazole are available. Other antifungal agents such as posaconazole, ravuconazole, caspofungin, and micafungin show promising results [2, 12, 13]. However, the application of azole derivative agents such as itraconazole may result in various side effects such as cardiac failure, mitochondrial dysfunction, or cardiomyopathy [14]. The research of the side effects of azole derivatives is required and the significance of the inhibitory effect should be analyzed taking into account the negative effects.

Another approach for growth inhibition of the pathogens, which allows addressing the side effect problem, is the photodynamic therapy [15, 16]. Application of the therapy in combination with antifungal agents of the azole family results in a less toxic treatment; however, more studies have to be performed to confirm and clarify the role of photodynamic therapy and its interaction with antifungal agents [15].

Electroporation or electropermeabilization is the increase of the cell membrane permeability by means of application of the pulsed electric field of controlled duration and intensity [17]. As an applicator usually a cuvette with integrated electrodes is used for* in vitro* studies and for* in vivo* various invasive multielectrode structures are applied, respectively [18, 19]. The cuvette or electrodes are connected as a load to the high power generators (electroporators) for high voltage pulse generation [19]. Depending on the treatment parameters such as pulse polarity, duration, amplitude, or repetitive frequency the efficacy and the extent of permeabilization are controlled [20]. The effect may range from temporary permeabilization of cell membrane to irreversible electroporation resulting in a nonthermal ablation of tissue [21]. Electroporation proved to be an effective drug delivery method used in electrochemotherapy, which is nowadays used in the treatment of cancer [22, 23].

Currently, the number of works focusing on electroporation of pathogens, especially* Candida,* is very limited [24, 25]. In our previous works we have shown that application of monopolar electric field pulses results in growth inhibition of* C. albicans* and has a potential as a synergistic less toxic method for treatment of this pathogen [25]. However, the effects of pulsed electric fields (PEF) on drug resistant species of* Candida* such as* C. krusei*,* C. guilliermondii,* or* C. lusitaniae* are yet to be studied. The influence of the pulse parameters on the treatment outcome was also not revealed previously.* C. lusitaniae* is an opportunistic yeast pathogen which can readily develop* in vivo* resistance to amphotericin B during the therapy [26] and therefore was chosen for the further experiments. In this work as an alternative antifungal treatment the electroporation method was evaluated. We presented novel experimental data of the dynamics of growth inhibition of the* C. lusitaniae* using monopolar, bipolar symmetrical, and bipolar asymmetrical electric field pulses.

## 2. Materials and Methods

### 2.1. Cells

The study was performed on* Candida lusitaniae* strain CL18 isolated from the skin of patients with clinical diagnosis of atopic dermatitis [27, 28]. Yeast cells were grown on the rich standard YPD medium (2% glucose, 2% peptone, 1% yeast extract, and 1% agar) for 48 hours at 30°C. Then, the cells were suspended in 1 M sorbitol (ROTH, Germany) to a concentration of 10^10^ CFU/mL. 80 *μ*L of the suspension was used for the pulsed treatment. After the electroporation the yeast cells were plated on the YPD agar. The colony forming units (CFU) were counted after 48 h of incubation at 30°C. No less than three independent experimental instances have been performed for each unique experiment.

### 2.2. Pulsed Power Setup

The bipolar ±1 kV, 100 A electric pulse generator developed in High Magnetic Field Institute, Vilnius Gediminas Technical University, has been applied in the study. The generator is capable of generating 1 *μ*s–10 ms square wave single and bursts of monopolar and bipolar pulses of variable duration and amplitude [29]. The 420 *μ*F capacitor array was used in the prototype for energy accumulation to ensure the square wave pulse waveform. As a load the electroporation cuvette with 1 mm gap between the aluminum electrodes has been used (BTX, Cuvette plus, Nr. 610, San Diego, USA).

**Figure 1:**
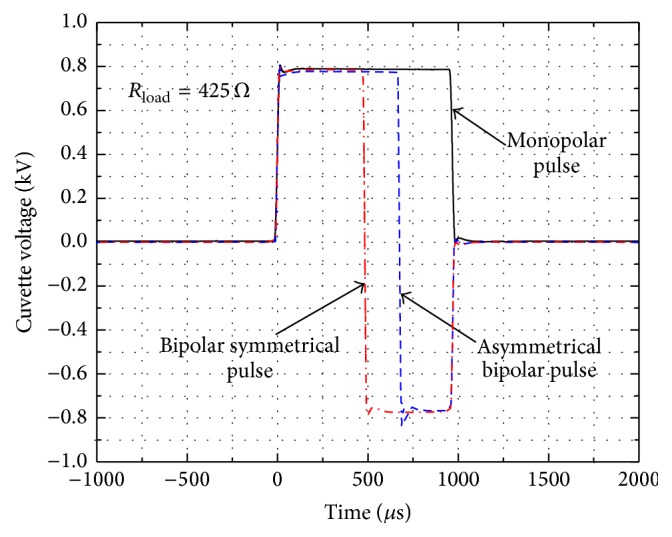
The waveforms of the generated monopolar, bipolar symmetrical, and bipolar asymmetrical pulses.

The waveforms of the generated monopolar, bipolar symmetrical, and bipolar asymmetrical pulses are shown in Figure 1.

During pulsing, depending on the load the high current is flowing through the cell buffer, resulting in thermal effects (Joule heating), which may distort or influence the experimental results. Since the aim of the study was to analyze the nonthermal effects of the electrical pulses, the study was limited to high impedance cell buffer (D-sorbitol, ROTH, Germany). A shunting resistance *R*_LOAD_ = 425 *Ω* was introduced in parallel to the cuvette in order to diminish the tail current effect during switching and ensure square wave pulse form [29].

The study of* C. lusitaniae* growth inhibition and permeabilization has been performed in a broad range of treatment parameters. The pulse duration was varied in a 20 *μ*s–1 ms range, while the electric field intensity was varied in the 2–8 kV/cm range (cuvette voltage of 0.2–0.8 kV).

### 2.3. Fluorescent Microscopy

For the fluorescent microscopy* C. lusitaniae* cells were harvested by centrifugation at 2000 rpm for 5 min and stained with 50 *μ*M propidium iodide for 5 min at room temperature. After the staining the cells were washed 3 times with PBS buffer, applied to polylysine coated slides, and immediately analyzed by fluorescent microscopy using 550 nm wavelength (Nikon Eclipse 80i, Japan). The cells were calculated manually; 300 cells were counted for each point. No less than three independent experimental instances have been performed for each unique experiment.

## 3. Results and Discussion

### 3.1. Growth Inhibition Dynamics

**Figure 2:**
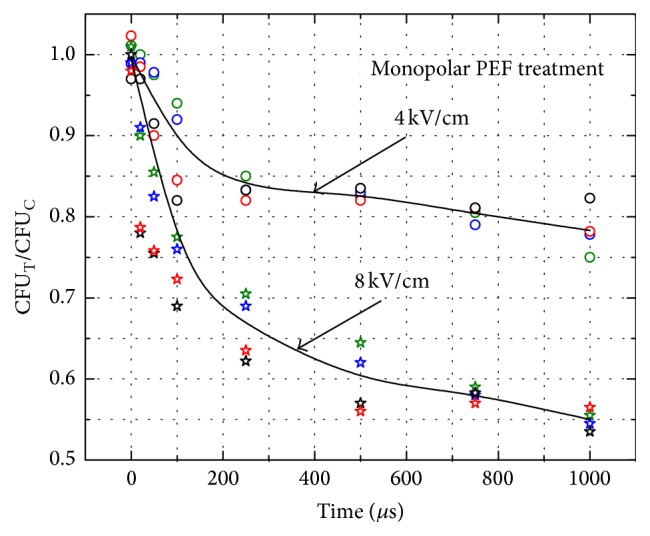
*C. lusitaniae* growth inhibition dynamics triggered by monopolar electric field pulses.

The* C. lusitaniae* have been subjected to 4 kV/cm and 8 kV/cm PEF. The exposure time has been controlled in the 20 *μ*s–1 ms range and the respective growth inhibition dynamics of the colonies have been evaluated by calculation of the colony forming units (CFU) after the 48 h of incubation. The CFU in the samples after the PEF treatment (CFU_T_) and the CFU in the control samples without treatment (CFU_C_) have been compared. The growth inhibition dynamics of the* C. lusitaniae* triggered by the monopolar electric field pulses of varied duration are shown in Figure 2.

Mean values for the given pulse durations were calculated from at least three independent experiments. The up to 44 ± 2% reduction of the CFU was achieved when the 8 kV/cm (1 ms) PEF treatment was applied. The 4 kV/cm pulse of the same duration resulted in a 22 ± 4% efficacy, respectively. The acquired PEF treatment efficacy increase was nonlinear for both treatment intensities. As it can be seen in Figure 2 the growth inhibition of the* C. lusitaniae* was more rapid in the 20–250 *μ*s pulse duration range, resulting in the up to 17 ± 2% efficacy for the 4 kV/cm pulse and 33 ± 4% for the 8 kV/cm, respectively. The CFU_T_/CFU_C_ mean difference between the 250 *μ*s–1 ms pulse treatments was not statistically significant in both the 4 kV/cm and 8 kV/cm cases (*P* > 0.05).

**Figure 3:**
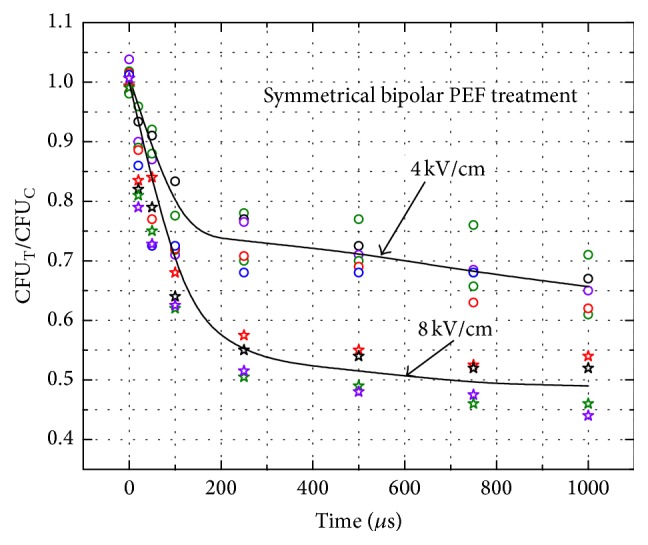
*C. lusitaniae* growth inhibition dynamics triggered by symmetrical bipolar electric field pulses.

Using the same methodology, the results of the bipolar symmetrical pulse PEF treatment were compared. The parameters of the bipolar pulses have been adjusted to match the duration and amplitude of the monopolar pulses (Figure 1). The total delivered energy of the bipolar pulses was equal to the energy of the respective duration of monopolar pulse (*E*_MP_ = *E*_BP_). The growth inhibition dynamics of the* C. lusitaniae* induced by the bipolar symmetrical pulses are shown in Figure 3.

Similar tendency of the growth inhibition of* C. lusitaniae* was observed during the bipolar pulse electroporation. In the 4 kV/cm instance a maximum treatment efficacy of 36 ± 6% was achieved (22 ± 4% for the monopolar pulse). The 8 kV/cm (1 ms) treatment resulted in the 51 ± 5% CFU reduction (44 ± 2% for the monopolar pulse). On average the bipolar pulses were more effective for irreversible permeabilization compared to the monopolar ones. However, the statistically significant difference (*P* < 0.05) was observed only in the 4 kV/cm, 1 ms treatment case.

**Figure 4:**
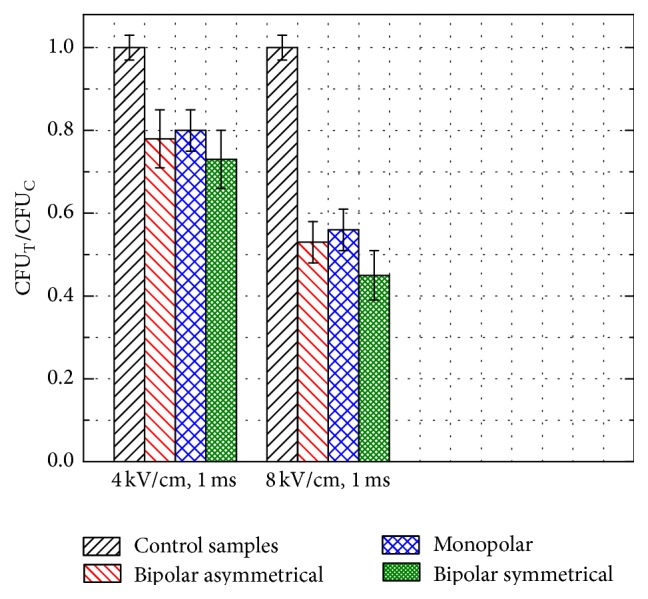
*C. lusitaniae* growth inhibition dynamics triggered by PEF pulses of varied shape. Columns represent the average ± SD of at least three independent experiments.

The asymmetrical bipolar pulses have been further introduced in the study. Taking into account the maximum inhibition rate during the monopolar and bipolar symmetrical pulses, the 1 ms pulse duration was used. The 8 kV/cm and 4 kV/cm PEF asymmetrical bipolar pulses (700 *μ*s positive, 300 *μ*s negative (Figure 1)) were applied. The experimental results featuring the dependence of* C. lusitaniae* CFU reduction on the electrical pulse shape are summarized in Figure 4.

The inhibition effect of the asymmetrical bipolar pulses of the identical energy (*E*_ABP_ = *E*_MP_ = *E*_BP_) showed no controversy. The average efficacy difference was within the standard deviation of the experiment. The increase of the inhibition rate due to the pulse shape was statistically significant only in the 4 kV/cm, 1 ms instance between the monopolar and bipolar symmetrical pulses (*P* < 0.05). Therefore, the electric field strength and the total pulse energy remain the most important parameters for definition of the treatment outcome.

### 3.2. Fluorescent Dye Uptake

**Figure 5:**
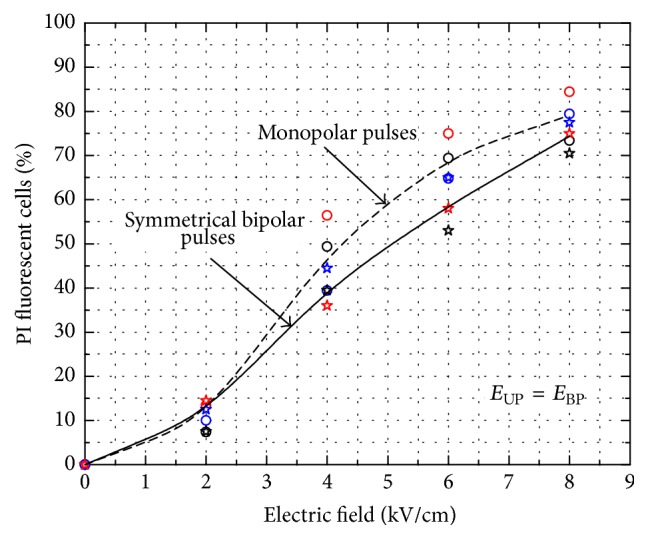
Dye uptake dynamics triggered by the monopolar and bipolar symmetrical electrical field pulses of 1 ms duration.

In order to evaluate the* C. lusitaniae* permeabilization rate dependence on the electric field strength, the propidium iodide uptake during a 1 ms pulse of 2–8 kV/cm was investigated. After the pulsed treatment the cells were stained and analyzed using fluorescence microscopy. The percentage of the propidium iodide uptake was calculated as the difference in the percentage of the fluorescent cells between the negative control (no PEF treatment) and the value in the treated sample. The results of PEF with monopolar and bipolar symmetrical pulses are presented in Figure 5.

As it can be seen in Figure 5 the increase of the electric field strength resulted in the increase of the number of the PI stained cells. Mean values for the given pulse amplitudes were calculated from at least three independent experiments. On average the bipolar pulses showed the slightly inferior permeabilization efficacy; however, the difference was not statistically significant (*P* > 0.05). The differences in the dye uptake between the treatments with various pulse shapes were within the standard deviation of the experiments.

During the 8 kV/cm pulse the number of the permeabilized cells was in the range of 79 ± 5%. However, the highest inhibition rate of 51 ± 5% was achieved (Figure 4), which implies that not all of the fluorescent cells were irreversibly electroporated.

**Figure 6:**
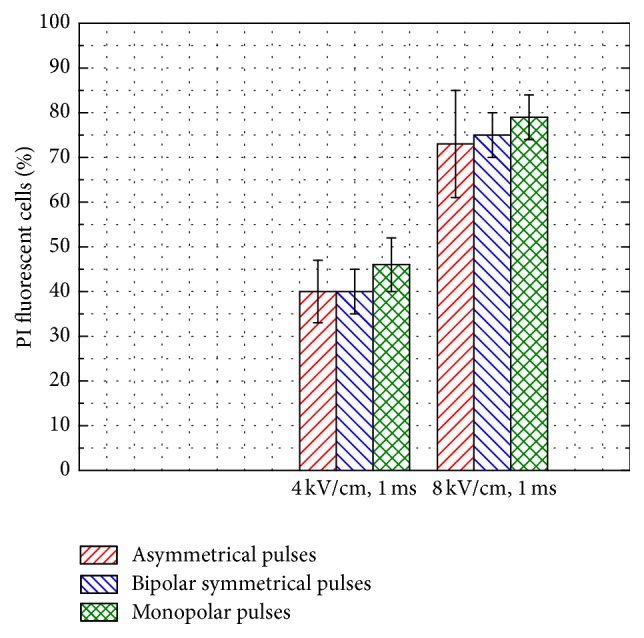
Dye uptake dynamics triggered by PEF pulses with varied shape. Columns represent the average ± SD of at least three independent experiments.

The 4 kV/cm and 8 kV/cm asymmetrical bipolar pulses have been further introduced in the study. The results summarizing the dependence of the propidium iodide uptake on the pulse shape are shown in Figure 6.

As it can be seen in Figure 6 the pulse shape influence on the experimental results is statistically insignificant (*P* > 0.05). The differences are within the standard deviation of the experiments. Similarly, as in the monopolar and bipolar symmetrical pulse cases, the number of the fluorescent cells during the asymmetrical pulse treatment was significantly higher (*P* < 0.05) compared to the inhibition efficacy (Figure 4).

### 3.3. Discussion

In this paper the comparison of the growth inhibition and membrane permeabilization of* C. lusitaniae* during the varied pulse shape electroporation is presented. As mentioned above, although* C. albicans* remains the most frequent yeast pathogen, an increasing proportion of nonalbicans* Candida* species has emerged. The biofilm-forming capacity of* Candida* species has been implicated as a potential virulence factor in the development of candidemia. Biofilm-associated* Candida* show uniform resistance to a wide spectrum of the currently available conventional antifungal agents, which implies that antimicrobial drugs that specifically target biofilm-associated infections are needed. It has been shown that some antifungals were able to cause damage to the biofilm structures of* Candida* species [13]. However, these antifungals should be used in high concentrations and species-specific differences in the susceptibilities of the biofilms formation have been observed [8, 10]. Therefore, in our work the main questions were how the different electrical pulse parameters (duration, shape, and electric field intensity) affect the inhibition of the pathogenic yeast cells and the integrity of the cell membrane. The results contribute both (1) to the electroporation field due to the comparison and analysis of the various pulse shapes and also (2) to the study of the* C. lusitaniae*, presenting novel data on the permeabilization dynamics of this pathogen.

To the best of our knowledge, this is the first experimental parametric analysis on the effects of the varied pulse shape (monopopolar, bipolar symmetrical, and asymmetrical pulses) electroporation on the* Candida* species. Nevertheless, the results have shown no controversy with the general electroporation studies and contribute to the research of the equivalent pulse parameters for PEF treatment. The experimental data indicate that during the single pulse electroporation the bipolar pulses on average result in a better inhibitory effect. However, it should be noted that the difference was statistically significant (*P* < 0.05) only during the 1 ms 4 kV/cm procedure. In the future works the study of the inhibitory effects of the bursts of the bipolar pulses should be addressed. Sano et al. through the finite element method simulation have shown that, by reducing of the delay between the consecutive bipolar pulses, it would be possible to achieve the doubling of the transmembrane voltage and, therefore, promote the efficacy of the treatment [30].

In our study the treatment of* C. lusitaniae* showed the inhibition efficacy of up to 51 ± 5% after the 8 kV/cm 1 ms bipolar pulse procedure. These results indicate that electroporation can be used as a tool to prevent or suppress biofilm formation in medical devices. Also the combination of the antifungal drugs with PEF may result in a more potent clinical method as an antifungal therapy to treat the skin infections, which are caused by the drug-resistive pathogens. The research of the effective treatment protocols should be performed.
